# Alcohol prevention at sporting events: study protocol for a quasi-experimental control group study

**DOI:** 10.1186/s12889-016-3145-3

**Published:** 2016-06-06

**Authors:** Natalie Durbeej, Tobias H. Elgán, Camilla Jalling, Johanna Gripenberg

**Affiliations:** Centre for Psychiatry Research and Education, Department of Clinical Neuroscience, Karolinska Institutet, & Stockholm Health Care Services, Stockholm County Council, Norra Stationsgatan 69, SE-113 64 Stockholm, Sweden

**Keywords:** Community-based intervention, Responsible beverage service, Licensed premises, LP, Blood alcohol concentration, BAC, Pseudo-intoxicated patrons, Alcohol prevention, Overserving, Football matches

## Abstract

**Background:**

Alcohol intoxication and overserving of alcohol at sporting events are of great concern, given the relationships between alcohol consumption, public disturbances, and violence. During recent years this matter has been on the agenda for Swedish policymakers, authorities and key stakeholders, with demands that actions be taken. There is promising potential for utilizing an environmental approach to alcohol prevention as a strategy to reduce the level of alcohol intoxication among spectators at sporting events. Examples of prevention strategies may be community mobilization, Responsible Beverage Service training, policy work, and improved controls and sanctions. This paper describes the design of a quasi-experimental control group study to examine the effects of a multi-component community-based alcohol intervention at matches in the Swedish Premier Football League.

**Methods:**

A baseline assessment was conducted during 2015 and at least two follow-up assessments will be conducted in 2016 and 2017. The two largest cities in Sweden are included in the study, with Stockholm as the intervention area and Gothenburg as the control area. The setting is Licensed Premises (LP) inside and outside Swedish football arenas, in addition to arena entrances. Spectators are randomly selected and invited to participate in the study by providing a breath alcohol sample as a proxy for Blood Alcohol Concentration (BAC). Actors are hired and trained by an expert panel to act out a standardized scene of severe pseudo-intoxication. Four types of cross-sectional data are generated: (i) BAC levels among ≥ 4 200 spectators, frequency of alcohol service to pseudo-intoxicated patrons attempting to purchase alcohol at LP (ii) outside the arenas (≥200 attempts) and (iii) inside the arenas (≥ 200 attempts), and (iv) frequency of security staff interventions towards pseudo-intoxicated patrons attempting to enter the arenas (≥ 200 attempts).

**Discussion:**

There is an urgent need nationally and internationally to reduce alcohol-related problems at sporting events, and it is essential to test prevention strategies to reduce intoxication levels among spectators. This project makes an important contribution not only to the research community, but also to enabling public health officials, decision-makers, authorities, the general public, and the sports community, to implement appropriate evidence-based strategies.

## Background

The relationship between alcohol and alcohol-related problems such as violence has been confirmed by several studies [1–3], and can be explained by various factors, e.g. the amount of consumed alcohol and the level of intoxication [4, 5]. In the mid 1990s, our research unit STAD (Stockholm Prevents Alcohol and Drug problems) developed a multi-component community-based Responsible Beverage Service (RBS) alcohol prevention programme in order to reduce heavy alcohol use, intoxication levels, and violence at licensed premises (LP) [6]. The programme comprised components such as community mobilization (cooperation with all important stakeholders), training of servers, stricter enforcement of existing alcohol laws, and policy work [7]. According to Swedish evaluations, the programme has been associated with reductions in levels of overserving to under-aged and pseudo-intoxicated patrons [6, 8], the number of alcohol-related violent crimes [7, 9] and emergency room visits [10], as well as reduced societal costs for alcohol-related violent crimes [11]. Since the mid-2000s, the programme has been disseminated and implemented in several Swedish municipalities. Altogether, research suggests that RBS programmes can be effective in reducing levels of overserving and alcohol-related problems in the community in both Sweden and other countries [12–14]. In 2015, STAD initiated a novel research project aiming to reduce intoxication levels among spectators at sporting events in Sweden. Heavy alcohol use and alcohol-related problems at sporting events, e.g., assaults, public disturbances, and vandalism, have attracted a great deal of media attention and also been commonly reported in both national and international research [15–18]. There are a number of studies that suggest a relationship between alcohol consumption and violence among spectators at sporting events (e.g., [19–21]). In Sweden, the situation escalated in 2002 and 2014, resulting in the deaths of two people. During the last few years, the matter has been on the agenda for Swedish policymakers, authorities and key stakeholders, with demands that actions be taken in order to reduce violence and other alcohol-related problems at these events. Similar concerns exist in many other countries. A promising strategy to reduce alcohol consumption and alcohol-related problems at sporting events is to implement multi-component local interventions, addressing for instance alcohol availability and pricing, stricter enforcement, and training of staff in RBS [22–24].

Despite the occurrence of heavy alcohol use and alcohol-related problems at sporting events, research on actual intoxication levels and levels of overserving in this setting is rather scarce. Studies conducted in the US have assessed Blood Alcohol Concentration (BAC) levels among spectators at baseball games and American football games and demonstrated that approximately 40 % tested positive for alcohol [25, 26]. The BAC levels ranged between 0.005 and 0.217 %, with a mean value of 0.057 among those who tested positive [25]. Furthermore, research has shown that many sport stadiums have a high likelihood of serving alcohol to obviously intoxicated patrons. More specifically, a US study, which used trained actors attempting to purchase alcohol while acting out signs of obvious intoxication, showed that overall sales rates were 74 % [27]. The method of using trained actors attempting to purchase alcohol while acting obviously intoxicated has been used in various studies and settings and can been regarded as valid for assessing levels of overserving [28–34].

To conclude, research suggests that alcohol use, intoxication and overserving are rather common features at sporting events. Community-based alcohol interventions at sporting events may be beneficial to reduce alcohol-related problems [22, 24, 30], for instance by decreasing intoxication levels and levels of overserving among spectators. To our knowledge, this has not been explored in previous research. The current study reports the methodology used in our research project aiming to explore whether a community-based alcohol intervention allocated to Swedish sporting events can reduce intoxication levels among spectators. Swedish football arenas serve as the primary research setting in this project.

### Objective and research questions

The aim of this study is to examine the effects of a multi-component community-based alcohol intervention at matches in the Swedish Premier Football League (SPFL). Specific research questions pertain to whether a community-based alcohol intervention will have an effect on a) intoxication levels among spectators attending the football matches and alcohol service to obviously alcohol-intoxicated patrons at LP (i) inside and (ii) outside the arena, and b) security staff intervention towards obviously alcohol-intoxicated patrons entering the arena. The findings are expected to yield information that can be used in order to develop intervention strategies to reduce intoxication levels and alcohol-related problems at football arenas as well as at other sporting events in Sweden.

## Methods/Design

This study uses a quasi-experimental control group design, where Stockholm (the largest city and capital of Sweden) is the intervention area and Gothenburg (Sweden’s second largest city) is the control area. Data is collected using three cross-sectional measurements, where the two study areas are followed over time.

### Setting

**Fig. 1:**
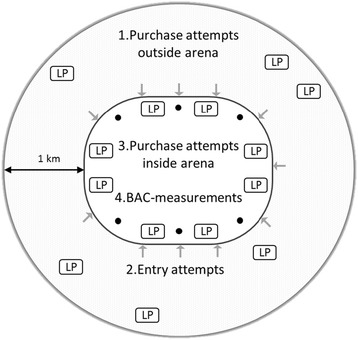
Schematic overview exemplifying the data collection setting

Data collection takes place before and during SPFL matches. The setting is football arenas hosting SPFL matches in Stockholm and Gothenburg, as well as an area defined as a radius of 1 km outside each arena (Fig. 1).

(1) Licensed premises (LP) within a radius of 1 km outside the arena are assessed using pseudo-intoxicated patrons attempting to purchase alcohol. (2) Pseudo-intoxicated patrons then attempt to enter the arena at the entrances (grey arrows). (3) Next, pseudo-intoxicated patrons attempt to purchase alcohol at the LP inside the arena. (4) The levels of intoxication among spectators are assessed through breath alcohol samples at six different strategically selected data collection spots inside the arena (black dots). To avoid that the Blood Alcohol Concentration (BAC) assessments influence the assessments using pseudo-intoxicated patrons (and vice versa), they are conducted on different match days.

In Stockholm, there are two arenas hosting SPFL matches. One arena has the capacity for 33 000 spectators and is home to two SPFL teams and the other arena is home to one SPFL team and has the capacity for 50 000 spectators. Both arenas are located just outside the city centre of Stockholm. Gothenburg has one arena located in the central part of the city, which is home to one SPFL team and has the capacity for 18 000 spectators. There are a number of LP in the close vicinity of all arenas.

At the arenas, there are several different LP (Fig. 1) where alcohol can be purchased. There are kiosks selling medium strength beer (3.5 % by volume) and bars and pubs selling all types of alcohol, including strong beer (> 4.5 % by volume) and hard liquor. The number of kiosks and bars/pubs varies by arena and also by the estimated number of visitors at a particular match, but can be as many as 16 kiosks and 10 bars and pubs. In addition, the arenas have VIP lounges available to sponsors etc., where alcohol, including hard liquor, is available.

In an attempt to reduce alcohol-related problems during football matches, the Swedish Elite Football Association, which is in charge of the SPFL together with the teams, does not schedule matches on Friday or Saturday nights, but rather on weekday evenings and in the daytime on weekends. Furthermore, at some high-risk matches, for instance local derbies, alcohol sales are limited to medium strength beer at the kiosks and bars/pubs.

At each arena there are a number of entrances (see Fig. 1) which are manned by 1-2 security guards who check tickets. In addition, there are uniformed police officers and licensed security staff within close reach of some entrances and also circulating around the outside of the arena. Inside the arenas there are civilian police officers and licensed security staff circulating around the arena.

### Participants

With regards to the BAC assessment, participants comprise spectators inside the arenas. Inclusion criteria are as follows: being a spectator inside the arena and being 16 years or older. Those 15 years or younger are excluded from the study.

With regards to assessments using pseudo-intoxicated patrons, participants comprise LP both inside and outside the arenas, as well as arena entrances. With regards to LP inside and outside the arena, the inclusion criteria are being open during the time of the event (in some cases, this means daytime), and for LP outside the arena, being located within a 1 km radius from the arena. Attempts are not made at LP considered to have too few patrons. All arena entrances open during the time of the event are included.

### Procedure

Prior to the initiation of data collection, all football clubs concerned, as well as the organizers of the events, were approached to gain approval for conducting BAC measurements during matches. They were also informed that an observational study was to be conducted; however, they were not informed about the use of pseudo-intoxicated patrons. All included arenas were visited at least once prior to data collection, in order to get an overview of the arenas and where to strategically place the research teams on days of data collection (see below). Data are collected on match days, which can be either weekdays (evenings) or weekends (daytime or afternoon). However, in order to avoid that the BAC assessment and the assessment using pseudo-intoxicated patrons influence the respective outcomes, these two data collection methods are scheduled to be conducted on different match days.

#### Level of intoxication using breath alcohol assessment

Female and male research staff are recruited and trained during a two-hour session prior to the initiation of data collection. On each match day, one project leader with overall responsibility and 18 research staff (including staff from STAD) meet up about two hours before kick-off. The research staff is divided into six data collection teams; each team thus consists of three staff members, where one has the responsibility to recruit participants, while the other two are responsible for data collection. It is important that the recruiter has an outgoing personality and that he/she is socially skilled. In order to include a variety of participants, the teams are strategically placed at different sections of the arena (Fig. 1), e.g., the supporter sections for the home team and the away team, the public sections, and the family section. Data collection starts about one hour before kick-off and continues during the first half of the match, during the half-time break, and about five minutes into the second half of the match.

Spectators are randomly selected and invited for study participation by the recruiter using a protocol where every third person who crosses an imaginary line, defined by each research team, is approached and invited to participate in the study. To minimize refusal rates, if the approached person belongs to a group, all members of that group are invited to participate [35, 36]. The invited person(s) is/are verbally presented with an informed consent statement; however, no signatures are required given the importance of maintaining confidentiality. If a person or a group of individuals refuse(s) to participate, the recruiter records them as drop-outs and takes note of gender and approximate age.

Individuals who accept study participation are given a glass of water to rinse their mouths, in order to obtain accurate readings from the alcohol breath test. The face-to-face interview is then initiated, followed by the alcohol breath test. The result from the breath analyser is instantly available and the participant is given information on his/her BAC level. If a participant expresses any concerns regarding his/her alcohol consumption, a written information sheet is handed out regarding where to find further information and where he/she can receive support. When data collection is completed, the project leader and research staff gather and fill in a protocol on how the data collection went, reasons for drop-outs etc.

#### Level of overserving or entries allowed using pseudo-intoxicated patrons

Eight professional male actors between 30 and 45 years of age have been hired as pseudo-intoxicated patrons and trained by an expert panel to act out a standardized scene of severe alcohol intoxication. The level of alcohol intoxication portrayed by the actors is the same as has been used previously [6], is considered a level of severe intoxication, and should lead to being denied service of alcohol or denied entrance to the arenas, in accordance with Swedish law. The expert panel consisted of a police officer, professional bartenders, a LP manager, an officer from the licensing board, a film and TV producer, and researchers and project leaders from STAD. All people involved had to sign a contract of secrecy.

On the days of data collection, one actor and one observer form a research team and a total of six research teams collect data during each football match. The role of the observer is to monitor each attempt by the actor and complete the accompanying protocol. About two and a half hours before each kick-off, the research teams and a project leader from STAD gather at a site close to the arena. At this time, the project leader informs the research teams which LP outside the arena are to be visited and in which order. Furthermore, tickets are handed out to each research team. A total of three different LP are visited by each research team, where the actor attempts to order a strong beer. Each team then makes attempts to enter the arena at two different entrances (one attempt per entrance). If the actor is allowed entrance at the first entrance, he enters and then as soon as possible exits the arena in order to make the second attempt. A third ticket is used without acting out the scene of intoxication in order to enter the arena. The entrances are selected so that all entrance sections are visited at least once. Once inside of the arena, each research team makes 3-7 attempts to purchase medium strength or strong beer, depending on availability and the number of kiosks/bars open at each section. Purchase attempts are made before the kick-off, during the match if there are other guests at the kiosk/bar, and during the half-time break.

When approaching a kiosk/bar or entrance, the actor acts out the standardized scene of severe alcohol intoxication. For instance, a number of different signs are portrayed by the pseudo-intoxicated actor before placing an alcohol order, such as staggering, slurred speech, inserting the credit card improperly, having problems focusing and fixing the gaze, and leaning over the bar. During the attempts at the entrances, the actor staggers, has a slurred speech, and fumbles when searching his pockets for the entry ticket. Once the actor judges that the serving or security staff have had ample time to observe the intoxicated behaviour, the actor places the alcohol order or shows the ticket to enter the arena. After completion of data collection, the project leader and research teams meet up to debrief how the data collection went.

### Pilot assessment

With regards to the BAC assessment, the first occasion of data collection is a pilot. Here, data collection is conducted according to the protocol as described above. If there are no major changes to be done to the protocol, the data is to be included in the baseline assessment. A pilot assessment using one pseudo-intoxicated actor is also conducted to ensure that the actors’ behaviours are standardized and to refine and test the observation techniques and the data collection instrument. Another aim with the pilot assessment is to test the feasibility of performing the standardized scene at sporting arenas during SPFL matches.

### Assessment

The study comprises one baseline assessment and at least two follow-up assessments. The baseline assessment was on-going during the football season 2015 (April through October) and was conducted prior to the intervention phase. Since the intervention will be initiated during the first half of 2016, follow-up assessments will be conducted about 18 and 24 months after the initiation of the baseline assessment. At the 18-month follow-up, only data from the BAC assessment will be collected. At the 24-month follow-up, data from both the BAC assessment and the assessment using pseudo-intoxicated patrons will be collected. It should be noted, however, that depending on funding, further long-term follow-up assessments may be conducted.

### Measures

#### Level of intoxication using breath alcohol assessment

The level of alcohol intoxication among spectators is measured using a breath analyser (Dräger Alcotest 6820, Drägerwerk AG & Co. KGaA, Germany), with the standardized sampling technique. This model is used by the police authorities in Sweden and older models have been used in other research studies (e.g., [37]). The instrument is programmed to reveal BAC levels in per mille, as opposed to breath alcohol concentration in mg/L. Data on demographics and recent self-reported alcohol use are assessed using a brief face-to-face interview developed by STAD. The interview comprises questions on gender, age, and alcohol use prior to entering the arena, as well as time and location at the arena for the assessment.

#### Level of overserving or allowed entries using pseudo-intoxicated patrons

Using the pseudo-intoxicated patrons, three types of data are generated: frequency of alcohol service to pseudo-intoxicated patrons attempting to purchase alcohol at LP (i) inside the arenas and (ii) outside the arenas, and (iii) frequency of security staff intervention towards pseudo-intoxicated spectators attempting to enter the arena. Furthermore, a number of possible confounding variables are also collected, as described below. For this purpose, a total of four protocols have been developed in order to record the outcomes. For the data collection at LP both outside and inside the arenas, there is one protocol each for the actors and the observers, respectively. The actors’ protocol focuses on the alcohol service outcome (served or not served) and how the service staff handles the situation, including type of server intervention. The observers’ protocol focuses on the service staff’s gender and age, crowdedness at the kiosk/bar/pub, number of intoxicated people, the overall orderliness, and the number of security staff at the kiosk/bar/pub.

For the purpose of data collection at the entrances of the arenas, two protocols have been developed, one each for the actors and observers, respectively. At the entrances, the main variable of interest is the outcome of the entry attempt, i.e., denied entry or not. The actors record if they are frisked and what types of intervention techniques are used by security staff. The observers record the number of security staff at the entrance and the presence of police officers, the number of persons in the entrance line, the overall orderliness at the entrance, and the number of intoxicated persons at the entrance.

### Intervention

In order to reduce the level of intoxication of spectators, a multi-component community-based alcohol prevention intervention is to be used. The alcohol intervention will utilize an environmental approach at the local level. Strategies selected will be based on needs assessments, in addition to baseline measurements, and could include strategies such as community mobilization, RBS training, training of security staff, policy work, media advocacy, and improved controls and sanctions. The intervention will subsequently be initiated in the intervention area and continue at least throughout the study period.

### Control area

The city of Gothenburg, i.e. the control area, has unrestricted access to intervention as usual. To our knowledge, there are no concurrent interventions specifically targeting security staff at the entrances to football arenas in Gothenburg, or alcohol service to obviously intoxicated patrons inside LP at football matches. Staff working at LP outside the arena may have participated in the RBS programme that has previously been developed at STAD and disseminated throughout Sweden. This will be documented during the intervention phase of the project.

### Sample size

To our knowledge, there have been no other similar studies reported in the research literature, in which BAC levels have been used to monitor changes due to a community-based intervention. We therefore hypothesize a reduction in BAC levels of about 0.015 %. An a priori calculation of the estimated sample size, using the software G*Power [38], reveals that BAC levels from a total of 1 400 participants (700 individuals in each study group) will be required at each data collection point in order to detect a reduction of 0.015 % among the spectators in the intervention area (power = 0.80, α = 0.05, 2-tailed). Thus, our aim is to include at least 4 200 participants in total, taking into account the three data collection points.

With regards to the assessment using pseudo-intoxicated patrons, assumptions made during power calculations are based on the logistic feasibility of the study and also on previous research conducted by our research group in the nightlife setting in Stockholm [6, 39, 40]. During a study of the effects of an RBS programme, the frequency of denial of alcohol purchase to intoxicated patrons at LP was 5 % (*n* = 92 attempts) at baseline and 47 % (*n* = 103 attempts) at the three-year follow up [6], which was statistically significant.

Given that our study will yield the same difference in proportions between the intervention and control area, a sample size of about 16 entry attempts in each area is needed (power = 80 %, alpha = 0.05). Allowing for smaller differences in proportions in the current study and the feasibility of conducting the data collection, the aim is to conduct at least 100 attempts at LP inside and outside the arenas as well as at the arena entrances, at each data collection point and study area, respectively. Thus, since this data comprises one baseline and one follow-up measurement, our aim is to include at least 200 attempts at LP inside the arenas, 200 attempts outside the arenas, and 200 attempts at the entrances.

### Statistical analyses

The study consists of cross-sectional data collected at three time points. Descriptive statistics will be computed in order to describe participants and settings. The statistical analyses will comprise methods to compare outcome measurements between the intervention and control area, at baseline and at subsequent follow-up assessments. The effects of the intervention will be estimated using effect size estimates.

## Discussion

This paper describes the protocol for a quasi-experimental control group study evaluating the effects of a multi-component community-based prevention intervention programme at football arenas in Sweden. The study uses two novel outcome measurements to assess changes over time. In order to assess the level of alcohol intoxication among spectators, breath alcohol samples, as a proxy for BAC levels, are collected on match days. To measure the degree to which severely intoxicated patrons are denied purchase of alcohol or allowed entry to football arenas, pseudo-intoxicated patrons enacting a standardized scene of severe alcohol intoxication are used. These techniques have been used by others in the nightlife setting [41–43], as well as by our research group, to evaluate the effects of an RBS programme [6, 44] and the Clubs against Drugs programme [39, 40].

### Strengths and limitations

There are a few limitations to this study. In order to draw conclusions about cause and effects due to an intervention it would have been desirable to use a randomized controlled trial design [45]. However, such a design is not feasible and this study instead uses a quasi-experimental design where the intervention area and a control area are as similar as possible, which enables valid comparisons between the study areas. Another limitation concerns the potential inclusion of intoxicated spectators with impaired cognitive functioning or judgment, contributing to biased responses during the face-to-face interviews.

There are a few strengths that should be mentioned. With regards to the BAC assessment, the intoxication levels among spectators are measured using biological samples and not self-reported measures of alcohol use. Thus, our data are not affected by under- or over-reporting, and thus the validity of the results is increased. All research staff are given formal training prior to data collection, which increases the reliability of the results. Moreover, the research teams are strategically placed at several sections of the arena, facilitating recruitment of spectators from the entire arena. Pseudo-patrons portraying alcohol intoxication have been used previously at sport stadiums [27] and by standardizing a scene with the help of an expert panel, it is to be expected that all actors perform the same intoxicated behaviour in all purchase and entry attempts. The level of alcohol intoxication portrayed by the actors is the same as has been used previously in other studies conducted by our research group [6, 44]. Furthermore, each attempt is monitored by an observer who completes a protocol, facilitating collection of detailed and accurate data. To our knowledge, this is the first study to utilize biological samples, i.e., breath alcohol levels, as an outcome measure to study the effects of a community-based alcohol intervention at sporting events. Finally, we are not aware of any previous study that has explored the effects of a community-based alcohol intervention targeting football matches at sporting arenas in Europe.

### Implication for practice and future policy

There is an urgent need to reduce alcohol-related problems at sporting events both in Sweden and internationally. One strategy to accomplish this goal is to reduce the overall level of intoxication among spectators. Since there is a lack of knowledge about the nature and extent of the problem and what prevention strategies are effective, this project proposes to contribute essential prevention research. One goal of the project will be to test promising strategies for prevention and intervention, to include components such as community mobilization, RBS, training of security staff, policy work, media advocacy, and improved controls and sanctions. To our knowledge, there are no studies in the research literature reporting on multi-component community-based alcohol prevention interventions targeting sporting events. This novel study therefore makes an important contribution not only to the research community, but also to public health officials, decision-makers, authorities, the general public and the sports community.

## Abbreviations

BAC, blood alcohol concentration; LP, licensed premises; RBS, responsible beverage service; SPFL, Swedish premier football league; STAD, Stockholm prevents alcohol and drug problems
